# Optimizing mycorrhizal fungi application for improved nutrient uptake, growth, and disease resistance in cardamom seedlings (*Elettaria cardamomum* (L.) Maton)

**DOI:** 10.1016/j.heliyon.2024.e39227

**Published:** 2024-10-10

**Authors:** Sarathambal Chinnathambi, Mohammed Faisal Peeran, Veeraraghavan Srinivasan, Sivasankaran Mukesh Sankar, Priya George

**Affiliations:** a ICAR-Indian Institute of Spices Research, Kozhikode, Kerala, India; b ICAR-IISR Regional Station, Appangala, Karnataka, India

**Keywords:** Arbuscular mycorrhizal colonization, Cardamom, Disease incidence, Nutrient uptake, Plant analysis, Structural equation modelling

## Abstract

This study evaluated the impact of various doses (5, 10, 15 g) and application sequences (1, 2, or 3 times at monthly intervals) of arbuscular mycorrhizal (AM) fungal inoculum on cardamom seedlings over two years (2020–2021 and 2021–2022). The results indicated that the dosage of AM inoculum had a more substantial effect on the seedlings than the application sequence. A 10 g dose significantly increased shoot length and dry weight, while three applications of 5 g each improved the number of fibrous roots. Although potassium uptake was not affected, phosphorus and calcium uptake were highest with the 10 g dose. Arbuscular mycorrhizal inoculation also enhanced phosphatase activity in the rhizosphere, with 5 g improving acid phosphatase and 10 g improving alkaline phosphatase activity. Disease incidence, including seedling rot was lower with the 10 g dose, and additional sequential applications did not further reduce disease. Structural equation modeling (SEM) revealed that AM colonization positively influenced dry weight through the number of fibrous roots, showing a strong relationship between AM dose, colonization, spore count, and mycorrhizal dependency. This study indicates that applying a 10 g dose of AM fungal inoculum can be particularly beneficial in agroecosystems for improving cardamom seedling growth, nutrient uptake, and disease resistance.

## 1 Introduction

Cardamom (*Elettaria cardamomum* (L.) Maton), known as the Queen of Spices, is a valuable spice native to the Indian Western Ghats and also grown in countries like Guatemala, Sri Lanka, and Tanzania [1]. In India, it is cultivated at elevations of 900–1400 m in Kerala, Karnataka, and Tamil Nadu, with the Indian Cardamom Hills being a major growing area. Besides its culinary use, cardamom is employed in traditional medicine for digestive and kidney disorders [2]. However, cardamom production faces challenges such as disease and poor planting material survival, necessitating improved propagation methods [3,4]. The use of arbuscular mycorrhizal (AM) fungi as a bioinoculant in potting mixtures is emerging as a sustainable solution to enhance seedling health, increase production, and reduce reliance on chemical fertilizers. Spice crops such as turmeric, ginger, black pepper and cardamom colonize endomycorrhizae fungus in their roots [5]. Furthermore, the use of AM fungus as a bioinoculant is being promoted as a method of increasing production while reducing the demand for chemical fertilisers [6,7].

Arbuscular mycorrhizal fungi are essential part of plant productivity, as their extraradical hyphae facilitate the absorption and distribution of nutrients in plants. Clearly, the introduction of AM fungi can substantially boost the levels of numerous macro and micronutrients, resulting in enhanced photosynthate production and, consequently, augmented biomass accumulation [8,9]. Among various nutrients, P and other micronutrients uptake have been found to be the utmost significant benefits of mycorrhiza. The presence of AM fungi in the soil can enhance the uptake of inorganic P from both soluble P pools and insoluble sources, such as rock phosphates. This occurs through local pH alterations and the production of organic acid anions, which act as chelating agents. In addition to this, mycorrhizal colonization can also impact the uptake of other essential nutrients such as K, Na, Mg, Cu, Fe, Zn and Mn by growing plants [10]. Arbuscular mycorrhizal fungi colonization possesses unique biological and physiological characteristics that make it promising for enhancing interactions between host plants and soil ecosystems. Additionally, research has shown that these fungi can increase the activity of important soil enzymes like phosphatase and dehydrogenase [11]. Recent studies highlight the significant role of arbuscular mycorrhizal fungi (AMF) beyond nutrient uptake in enhancing plant resilience under stress. Sheteiwy et al. [12] showed that *Rhizophagus irregularis* improved growth and stress tolerance in plants under beryllium stress, indicating its potential to mitigate heavy metal contamination. Arbuscular mycorrhizal fungi (AMF), such as *Rhizophagus irregularis Glomus monosporum* and *Funneliformis constrictum* enhance water use efficiency and mycorrhizal dependency at critical maize growth stages, potentially boosting yields under drought and salinity conditions [[13], [14], [15]].

Interestingly, plant protection chemicals such as fungicides and herbicides will not affect the growth of mycorrhizae [16]. Arbuscular mycorrhizal fungi influence their host plants in various ways beyond just improving nutrition. These beneficial fungi can affect the plant physiology and defensive chemicals [17]. Such changes may boost resistance to plant antagonists like nematodes and pathogens [18]. There have been various suggested mechanisms proposed to explain mycorrhizal-induced plant resistance, including priming plant phytohormonal defense pathways [19], strengthening cell wall defenses [20] and increasing production of defensive chemicals [21]. The ability of various mycorrhizal species to infect and benefit plants can vary depending on how well the mycorrhizal spores suit the host plant and soil environment. The quantity of starter inoculum needed for maximum inoculation is critical because the number of spores per plant depends on how effectively and infectively the spores can colonize [22]. The optimum level of mycorrhizal inoculum dose is important for agricultural plant seedlings because mycorrhizal fungi develop a symbiotic relation with plant roots, which can enhance plant growth and nutrition. However, the efficiency of mycorrhizal inoculation is affected by a variety of parameters, including the type of mycorrhizal fungus employed, the dosage utilized, the individual crop and soil conditions. The advantages to plant development and nutrition may be restricted if the dosage of mycorrhizal inoculation is too low. As a result, the optimum mycorrhizal dose for seedling crops must be carefully considered depending on parameters such as soil type, crop variety and nutritional requirements.

Singh et al. [23] demonstrated that the growth and fitness of Indian coleus cuttings (*Coleus forskohlii*) can be improved through colonization of their adventitious roots by AM fungi. However, it should be noted that one significant limitation in several studies examining the impact of AM fungi on seedling growth performance is that they are frequently carried out in disinfected soils or soilless substrates. Therefore, the main focus of this study is to optimize the dosage of arbuscular mycorrhizal (AM) fungi to enhance the growth, nutrient uptake, and disease resistance of cardamom seedlings under natural conditions. We hypothesize that varying doses of AM fungi will have a dose-dependent impact on these parameters, with an optimal dose leading to the best growth outcomes. Additionally, we hypothesize that different doses of AM fungi will significantly influence enzyme activity in the rhizosphere soil, contributing to the overall health and development of cardamom seedlings.

## 2 Materials and methods

### 2.1 Experimental design and plant material

The experiment was conducted over two years, from 2020 to 2022, in the shelter house at the ICAR-Indian Institute of Spices Research Regional Station in Appangala, Madikeri, Karnataka, India. The coordinates of the experimental area were 12°26′N Latitude, 75°45′E Longitude, and 920 m above mean sea level. This region experiences a temperature range of 12–30 °C, an annual rainfall of 3000 mm, and relative humidity levels ranging from 80 percent to 95 percent. Ninety-day-old, healthy transplanted cardamom seedlings (Variety: Appangala-1) at the three-leaf stage were used in the study. To assess the impact of AM inoculation in natural conditions, the experiment used an unsterilized potting mixture. Polythene bags with a capacity of 1 kg were used for the pot culture experiment. It consisted soil: sand: farm yard manure (1:1:1) and initial nutrient composition was analysed as per the standard procedures and were pH 6.7, organic carbon 2.0 percent, N 119 mg/kg, P 80.5 mg/kg, K 152.5 mg/kg, Ca 533 mg/kg, Mg 139 mg/kg, Mn 22.4 mg/kg, Fe 37 mg/kg, Cu 3.8 mg/kg and Zn 9.6 mg/kg. The treatment details were given as per Table 1. All the treatments were replicated five times.

**Table 1:** Experimental design for standardization of dose and application schedule of arbuscular mycorrhizal inoculum.

Dose	Control	Single application (30 days)	Two-Time application (30, 60 days)	Three-Time application (30, 60, 90 days)
**0 g**	D0S0			
**5 g**		D1S1	D1S2	D1S3
**10 g**		D2S1	D2S2	D2S3
**15 g**		D3S1	D3S2	D3S3

### 2.2 AM fungal inoculum preparation and application to cardamom seedlings

The AM fungi *Rhizophagus* sp*.* (MN710507) inoculum was prepared using vermiculite as a carrier. The 1 g inoculum contained 10 infective propagules, including hyphae, spores, and mycorrhizal roots. This was standardized by using direct microscopic counting of spores and hyphae. The inoculum was then mixed with the potting mixture according to the respective treatments before planting the cardamom seedlings.

### 2.3 Plant growth analysis

In the early stages of plant growth, AM symbiosis can enhance plant growth and development. Since, to study the initial responses of AM inoculation on cardamom seedlings, a different dose of AM was inoculated as per the treatments and samples were analysed at the end of 3rd month. The entire plant was uprooted and examined for growth parameters like shoot length, root length, number of leaves, number of fibrous roots and total biomass on dry weight basis [18].

### 2.4 Mycorrhizal parameters

To determine the percentage of AMF colonization, roots were cleared in 10 percent KOH and stained with 0.05 percent trypan blue in lactophenol following the method described by Phillips and Hyman [24]. Microscopic examination at 10× magnification was then used to estimate the colonization percentage. The degree of mycorrhizal colonization was calculated using the following formula:

$$\text{Per}\;\text{cent}\;\text{of}\;\text{mycorrhizal}\;\text{colonization}=\frac{\text{Number}\;\text{of}\;\text{root}\;\text{sections}\;\text{colonized}}{\text{Total}\;\text{number}\;\text{of}\;\text{root}\;\text{sections}\;\text{observed}\;}\;x\;100$$

The extraction of AM spores was carried out using a wet sieving and decanting method [25] as follows. Firstly, 50 g of soil sample was mixed in 500 mL of water in a beaker. Following a 1 h period, the contents of the beaker were decanted through a series of sieves, arranged in descending order from 600 μm to 37 μm in size. The resulting sievings were collected in a petriplate and the spores were counted under a stereo zoom microscope (Nikon, SMZ-U).

“Mycorrhizal dependency (MD) is the degree of plant growth change associated with arbuscular mycorrhizal (AM) colonization” and it was measured based on formula of Plenchette et al. [26]:

$$\text{MD}\;\left(\%\right)=\frac{\text{Dry}\;\text{weight}\;\text{of}\;\text{AM}\;\text{inoculated}\;\text{plant}\;‐\;\text{Dry}\;\text{weight}\;\text{of}\;\text{AM}\;\text{un}\;\text{inoculated}\;\text{plant}\;}{D\text{ry}\;\text{weight}\;\text{of}\;\text{AM}\;\text{inoculated}\;\text{plant}\;}\;x\;100$$

### 2.5 Plant nutrient uptake and soil phosphatase activity

For the nutrient uptake analysis, the plants were oven-dried at 60 °C and ground into a fine powder with a mixer grinder. The Kjeldahl method, as described by Nelson and Sommers [27], was employed to assess nitrogen (N) uptake. For the estimation of P, 1 g of the powdered plant sample was digested with a nitric acid (HNO_3_) and hydrochloric acid (60 percent HCl) mixture at a ratio of 9:4 v:v and analysed using a spectrophotometer at 660 nm [28]. The atomic absorption spectrophotometer (Varian AA 240FS) was used to estimate exchangeable K, Ca, Mg, and micronutrients Fe, Mn, Zn and Cu as per the method described by Thomas [29].

One gram of soil was placed in a 50-mL Erlenmeyer flask. To this, 250 μL of toluene and 4 mL of modified universal buffer (pH 6.5 for acid phosphatase or pH 11 for alkaline phosphatase) were added [30]. Subsequently, 1 mL of p-nitrophenyl phosphate solution was mixed in, and the flask was sealed with a rubber stopper. The mixture was incubated at 37 °C for 1 h. After incubation, 1 mL of 0.5 M CaCl_2_ and 4 mL of 0.5 M NaOH were added, mixed, and then filtered through Whatman no. 2 filter paper. The amount of p-nitrophenol released was estimated spectrophotometrically at 420 nm (Shimadzu, UV-1800).

### 2.6 Seedling rot disease incidence

In another pot experiment, cardamom seedlings inoculated with AM fungi were sequentially inoculated with *Pythium vexans* (PV APG2), *Rhizoctonia solani* (RS APG2), and *Fusarium oxysporum* (FO APG5). The AM inoculum application rate was as per section 2.1 per 1 kg of potting mixture. For uniformity in the experiment the fungal pathogens were inoculated 30 days after the first AM inoculation. *P. vexans* cultures, aged 72 h, were carefully excised from the growing edges. These culture samples were then transferred into Petri plates containing sterilized distilled water. Following this, they were incubated for a period of 24 h to stimulate sporulation. For each polybag, a total of 10 of these incubated discs (5 mm size) were employed as inoculums. *R. solani* and *F. oxysporum* were cultured in a sand-to-maize medium for challenge inoculation at a ratio of 10:1, and an appropriate amount of water was added to achieve the desired moisture level. Subsequently, 100 g of this mixture were transferred into a 250 mL conical flask, securely sealed and subjected to two rounds of sterilization at 121 °C for 1 h each. 5 mm discs from 5-day-old cultures of both *R. solani* and *F. oxysporum* were separately transferred into sterile sand-to-maize media in conical flasks. These cultures were then incubated at room temperature (24 ± 1 °C) for a period of 14 days. Each treatment was challenged with 10 g of inoculum, containing 3.5 × 10^6 CFU/g, that was pre-treated with AM fungi. All the treatments were replicated five times and each replication consisted of six seedlings.

The assessment of disease incidence was done based on the number of infected plants observed after a 15 days period of inoculation [31].

$$\text{Percent}\;\text{disease}\;\text{incidence}=\frac{\text{Number}\;\text{of}\;\text{seedlings}\;\text{wilted}\;}{\text{Total}\;\text{number}\;\text{of}\;\text{seedlings}\;}\;x\;100$$

### 2.7 Statistical analysis

The effect of AM inoculation dose, (with 3 levels) and AM inoculation sequence of application (with 3 levels) were determined by two factor factorial analysis of variance (ANOVA), and the Tukey HSD (Honestly Significant Difference) test at the 5 percent level was used for multiple comparisons among mean values using KAU GRAPES 1.0.0. (General Rshiny Based Analysis Platform Empowered by Statistics) developed by Kerala Agricultural University, India [32]. Multivariate trait association were summarized with Pearson correlation (r) coefficients using the R function ‘corrApp (.)’. In order to identify various key variables associated with P uptake and dry weight of plants upon AM inoculation, a structural equation model (SEM) was applied. With the aim of quantify the multivariate causal network in which AM dose, P, Zn, Mn, Mg uptake, spore count, colonization, mycorrhizal dependency, dry weight (DW), fibrous roots (FR), shoot length (SL), alkaline phosphatase (AlP) were all involved. FR, colonization, AlP and PH were considered direct factors, while Zn, Mn, Mg uptake, spore count, mycorrhizal dependency was considered indirect. Structural equation modelling is a multivariate statistical analysis technique that is used to analyze structural relationships [33]. The technique used in this analysis can be viewed as a combination of factor analysis and multiple regression analysis, enabling the estimation of multiple and interrelated dependencies within a single analysis. It is important to note that the double-headed arrows in the model represent the covariances among variables, while the coefficients associated with directed paths between two variables are partial regression coefficients. Also note that these coefficients do not represent the bivariate correlation between the two variables. To ensure the comparability of coefficients from different pathways, all selected variables were standardized to have a mean of 0 and a standard deviation of 1 prior to the SEM analysis. The R software platform was used to conduct the SEM [34] using R package ‘lavaan’ package [35]. The SEM fit was tested using p-values and χ2 values, with high p-values (P > 0.05) and small χ2 values indicating that the data was well-suited to the model. The overall model fit was evaluated using the adjusted goodness-of-fit statistic (AGFI >0.90) and the standardized root mean square residual (SRMR <0.05), as suggested by Hooper et al. [36]. The detailed script for the analysis can be found in Supplementary file S1.

## 3 Results

Based on the two year pooled data analysis, AM inoculum doses significantly influenced the variables such as root length, number of fibrous roots, dry weight, spore count, colonization, mycorrhizal dependency and nutrient uptake (P, Ca, Fe, Mn, Zn, Cu) soil enzymes (acid and alkaline phosphatases) and disease incidence. Sequences of AM inoculum application was significantly influenced the variables such as shoot length, number of leaves, mycorrhizal dependency, dry weight, nutrient uptake (N, P and Mg). Dose by sequence interaction significantly influenced the following variables such as number of leaves, mycorrhizal dependency and nutrient uptake (N, Fe, Mn, Zn) (Table 2).

**Table 2:** Significance level (P value) for plant growth, mycorrhizal parameters, nutrient uptake enzyme activities and disease incidence obtained from two factorial ANOVA.

Source pf variation	DOF	SL (cm)	RL (cm)	NOL	FR	DW (g)	SC (50 g sample)	Col (%)	MD (%)	N (g/plant)	P (g/plant)	K (g/plant)	Ca (g/plant)	Mg (g/plant)	Fe (mg plant)	Mn (mg plant)	Zn (mg plant)	Cu (mg plant)	AcP (μmol PNP/g soil/h)	AlP (μmol PNP/g soil/h)	PDI
Dose (D)	2	0.002	0.01	NS	0.03	0.007	<0.001	<0.001	<0.001	NS	<0.001	NS	0.00	NS	<0.001	0.00	0.04	0.002	<0.001	<0.001	0.001
Sequence (S)	2	0.01	NS	0.003	NS	0.001	<0.001	<0.001	<0.001	0.015	<0.001	NS	NS	0.00	NS	0.005	NS	NS	0.001	<0.001	NS
D ∗S	4	0.03	NS	0.013	0.005	NS	<0.001	<0.001	0.001	0.039	NS	NS	NS	NS	<0.001	0.003	<0.001	<0.001	NS	NS	NS

DOF: Degrees of freedom, SL: Shoot length, RL: Root length, NOL: No of leaves, FR: Number of fibrous roots, DW: Dry weight, SC: Spore count, Col: Colonization, MD: Mycorrhizal dependency, AcP: Acid phosphatase, AlP: Alkaline phosphatase, PDI: Percent disease incidence, significance at p < 0.05, NS: Non significant.

### 3.1 Arbuscular mycorrhizal (AM) fungi application rates on growth and mycorrhizal parameters of cardamom seedlings

**Fig. 1:**
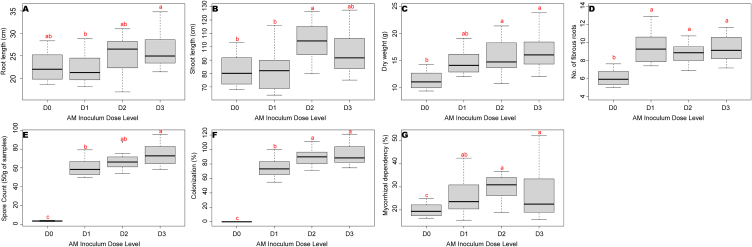
Plant growth parameters and Mycorrhizal parameters A). Shoot length, B). Root length, C). Dry weight and D). Number of fibrous roots, E). Spore count F). Colonization percentage and G) mycorrhizal dependency percentage as influenced by different rates of AM application.

The pooled analysis showed that the shoot length and root length was significantly increased with 102.2 cm and 27.95 cm in the treatment D2 (10 g) and D3 (15 g) respectively (Fig. 1A and B). Similarly, in dry weight of seedlings, at par results were observed with 10 g (15.81 g) and 15 g (16.8 g) of inoculum doses (Fig. 1C). Whereas 5 g of inoculum with three times application was helpful for the improvement of fibrous root numbers (9.55) in cardamom seedlings (Fig. 1D). Nevertheless, the AM dosage was not significantly influenced the number of leaves in cardamom seedlings. Among the plant growth parameters, the dry weight and number of leaves were positively influenced by the sequence application of inoculum.

Mycorrhizal parameters such us spore count and colonization percent were mainly influenced by the AM dosage. Significant number of spore load (73.0 spores/50 g substrate) and percentage of root colonization were observed in D3 (15 g) treatment (Fig. 1E and F). Mycorrhizal dependency percent was significantly influenced by the dose and sequence interaction on the cardamom seedlings. It was recorded between 20 and 39 percent of inoculated cardamom seedling with the various doses and sequences (Fig. 1G). Overall results showed that the AM inoculum doses enhanced shoot length, root length, and dry weight, with multiple applications boosting fibrous root development. Higher doses also increased mycorrhizal colonization and spore load, showing a strong dose-effect relationship.

### 3.2 AM fungi application rates on nutrient uptake and soil enzymes of cardamom seedlings

**Fig. 2:**
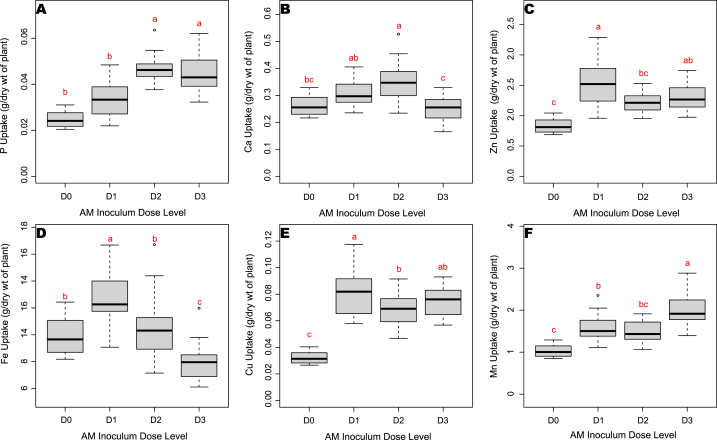
Nutrient uptake A). P, B) Ca, C). Zn, D). Fe, E). Cu, F). Mn as influenced by different rates of AM application.

Pooled analysis revealed that the nutrient uptake was positively correlated with the mycorrhizal treatments in cardamom seedlings. Particularly, the dose of inoculum significantly influenced the P, Ca, Fe, Mn, Zn, Cu content of the cardamom seedlings except that of N and Mg. As expected, P uptake was significantly increased in the AM inoculated plants D2 and D3 (0.05 g/dry wt) and both the dose and sequence of application significantly influenced the P uptake of cardamom seedlings (Fig. 2A and B). In contrast, N and Mg contents were influenced by the sequence of the AM application with uptake of 0.26 g/dry wt and 0.085 g/dry wt respectively. K uptake was not influenced by the AM inoculation. Uptake of Ca was high in treatment D2S2 with 0.415 g/dry wt (Fig. 2C).

Fe, Zn and Cu uptake also positively improved with five g dosage of inoculum, whereas, Mn required 15 g doses of inoculum for its improved uptake (2.2 mg/dry wt) (Fig. 2D–F). For majority of the micronutrients, 5 g of inoculum dose was ideal for improved uptake *viz*., Fe (13.7 mg/dry wt), Zn (1.77 mg/dry wt) and Cu (0.09 mg/dry wt) uptake under AM inoculated treatments.

**Fig. 3:**
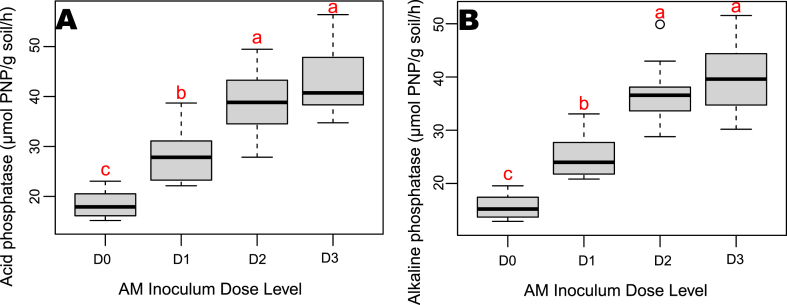
a) Acid phosphatase and b) alkaline phosphatase activity influenced as by different rates of AM application.

In case of soil enzymes activity, pooled analysis showed that both 15 g and 10 g inoculum doses were optimum for maximum enzyme activity (Fig. 3A and B). Among the AM applied treatments, the highest acid phosphatase (44.6 μmol PNP/g soil/h) and alkaline phosphatases (39.25 μmol PNP/g soil/h) activities were observed with treatment D2S1 and D2S2 respectively.

The pooled analysis showed that AM inoculum doses positively affected nutrient uptake, with higher doses improving phosphorus, calcium, and micronutrient levels, while 5 g was ideal for most micronutrients. Soil enzyme activities were maximized with 10 g and 15 g doses, with the highest acid and alkaline phosphatase activities found in specific treatment combinations.

### 3.3 AM fungi application rates on disease incidence of cardamom seedlings

**Fig. 4:**
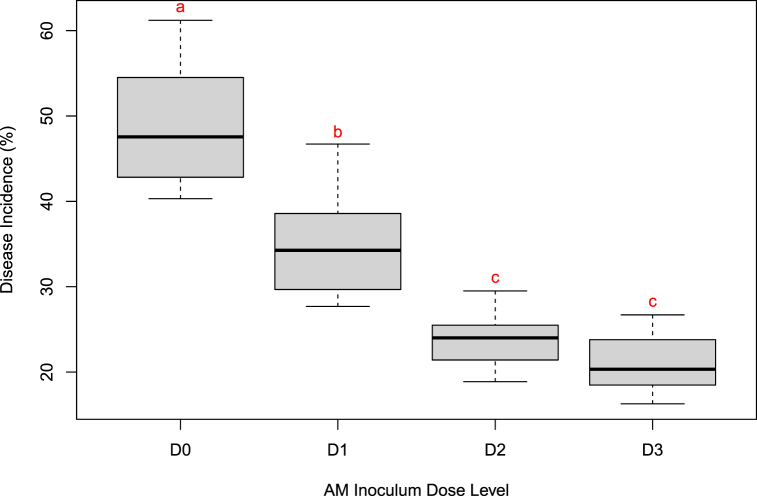
Percent disease incidence as influenced by different rates of AM application.

The disease incidence recorded upon fifteen days post challenge inoculation showed maximum incidence in control (49.28 PDI), followed by treatment D1 (5 g). The treatment D2 (10 g) and D3 (15 g) showed reduced disease incidences and found to be statistically on par. Irrespective of number of times of application, the initial dosage had the maximum influence over the disease control. The reduced disease incidence in treatment D2 indicates that, initial application of 10 g of AM inoculum is sufficient and further repeated application is not influencing the disease incidence statistically (Fig. 4). The study found that a 10 g dose of AM inoculum effectively reduced disease incidence in cardamom seedlings, with no additional benefit from further applications.

### 3.4 The relationship between mycorrhizal parameters and growth of cardamom seedlings

The preliminary analysis of the association of variables studied *viz*., Pearson correlation suggested a positive significant correlation exists, a positive significant correlation among most of the variables except the significant negative correlation found in the case of AM dose with iron uptake (r = 0.5). The AM dose showed a strong association (r > 0.70) with spore count (r = 0.73), percentage mycorrhizal colonization (r = 0.73), acid phosphatase (r = 0.80), and alkaline phosphatase (r = 0.80), moderate association (0.4 <r < 0.8) with dry weight (r = 0.47), P (r = 0.64), and Mn uptake (r = 0.58), and a weak association (r < 0.30) with AMF sequence of application (r = 0.36), fibrous root (r = 0.29), Mg (r = 0.34), and Cu uptake (r = 0.30). Similarly, the AM sequence of application showed a significant strong positive association with Cu (r = 0.71), moderate association with fibrous root (r = 0.43), spore count (r = 0.54), percentage mycorrhizal colonization (r = 0.59), P (r = 0.49) and Zn uptakes (r = 0·43), and alkaline phosphatase (r = 0.40) and a weak association with Mn uptake (r = 0.25) and acid phosphatase (r = 0.30). With respect to P uptake, dry weight (r = 0.76), spore count (r = 0.73), percentage mycorrhizal colonization (r = 0.78), and acid (r = 0.87), and alkaline phosphatase (r = 0.89) showed strong positive associations.

**Fig. 5:**
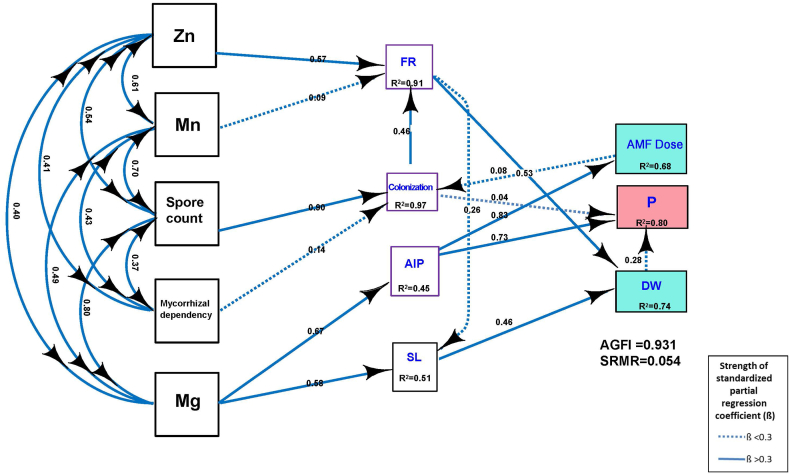
The structural equation model (SEM) showing the relationships among AM dose, P, Zn, Mn, Mg uptake, spore count, colonization, mycorrhizal dependency, dry weight (DW), fibrous roots (FR), shoot length (SL), alkaline phosphatase (AlP). The width of the arrows indicates the strength of the standardized path coefficient.

Further, structural equational modelling (SEM) was performed to assess the extent of the direct and indirect effects of explanatory variables on mycorrhizal parameters and other growth traits of cardamom seedlings (Fig. 5). SEM exhibited a reasonable fit based on our hypothesis with AGFI = 0.93 and the SRMR = 0.05. The dry weight gained (DW), alkaline phosphate enzyme activity (AlP) together with colonization accounted for nearly 80 percent of the variance (R^2^ = 0.80) in P uptake in the plant due to AM inoculation. P uptake was directly effected by dry weight (β = 0.74, P < 0.05), and alkaline phosphatase (β = 0.45, P < 0.05). activity and showed a weak association with mycorrhizal colonization (β = 0.97, P < 0.05). AlP was identified as a significant direct driver of P uptake with the path coefficients of 0.73 and was indirectly influenced by Mg uptake.

The positive indirect effects from spore count (β = 0.90, P < 0.05), mycorrhizal dependency (β = 0.14, P < 0.05), and dose of AM (β = 0.53, P < 0.05), explained almost 97 percent variance (R2 = 0.80) in colonization. On the other hand, colonization was strongly influenced by the number of fibrous roots (FR), (β = 0.14, P < 0.05). Other than colonization, Zn and Mn uptakes positively influenced FR contributing about 91 percent variance. Mg uptake observed to beindirectly influencing dry weight through shoot length. FR also showed a positive influence towards SL together explaining 50 percent of the variation in SL.

The analysis revealed strong positive correlations between AM dose and key variables like spore count and enzyme activities, with structural equation modeling indicating that dry weight and alkaline phosphatase significantly drive phosphorus uptake, while spore count and mycorrhizal dependency strongly influence colonization.

## 4 Discussion

Several critical factors, *viz*., ability of the introduced AM fungi to survive in the new habitat and outcompete native AM fungi, must be examined before using arbuscular mycorrhizal (AM) inoculum in agricultural applications. They must also be able to create symbiotic relationships with host plants in the field. For most of the crop species, selecting an optimum inoculum dosage is critical for improving plant development. The inoculum must contain a sufficient number of infective propagules that can survive and multiply after being introduced to field environments. When these features are taken into account, AM inoculum can be applied in a way that make best use to crop species by improving nutrition uptake, growth, and yield [37]. The main factor influencing the plant's growth was the dosage of AM inoculum, with three different levels being tested (5 g, 10 g, and 15 g). Out of these dosages, it was found that applying 10 g of AM was the most effective in achieving maximum growth for cardamom seedlings. In contrast to the dosage, application sequence did not significantly affect most of the growth parameters of the seedlings. These findings are consistent with previous studies reported that mycorrhizal inoculation was beneficial in improving the seedling growth, dry weight and P uptake of Vazhukka cultivar of cardamom seedlings under nursery conditions [38]. In our study, AM application can also improve shoot length and increase root biomass of cardamom seedlings [39,40]. In contrast to our results, reports have also shown that the effect of AM on biomass production is more noticeable in the roots of the plant than in the above-ground parts, such as stems and leaves, in black pepper cuttings [19].

**Fig. 6:**
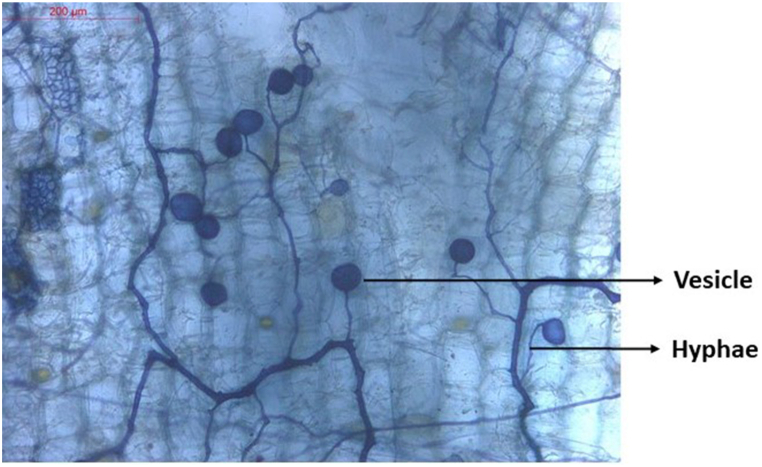
Colonization of arbuscular mycorrhizae in cardamom roots.

Khan et al. [41] and Rehman et al. [42] found that combining PGPR and AMF significantly enhances drought tolerance in wheat and maize. *Bacillus amyloliquefaciens* with *Rhizophagus irregularis* improved water uptake in wheat, while *Acaulospora laevis* with Bacillus subtilis boosted root colonization, nutrient uptake, and yield in maize under drought conditions. The observed significant variation in root colonization across different doses is evidence of the inoculum's ability to effectively colonize and beneficially influence the host. AM structures, including hyphae, arbuscules and vesicles were observed in the roots of inoculated seedlings (Fig. 6). Percent root colonization ranging from 72 to 95 were observed. Furthermore, we found that the level of root colonization was a main factor affecting the efficiency of AM fungi to increase plant growth parameters in cardamom seedlings [43]. The reliance of a crop species on mycorrhizal association, known as mycorrhizal dependency, is an innate trait that determines how it will respond to inoculation with arbuscular mycorrhizal fungus [44]. Mycorrhizal symbioses are widely recognized for their importance in nutrition uptake and ionic transport, particularly in P uptake. This mainly due to the mycorrhizal fungi exude organic acids and other chemicals that help dissolve P, Fe, and other nutrients that are immobilized in soil particles. By unlocking essential nutrients, the fungi greatly expand the volume of soil from which plants can draw resources. This is a key mechanism by which mycorrhizal associations improve plant nutrition and growth [10].

In our study, significant uptake of the P and Ca were noticed with 10 g of optimal dose of AM inoculation. Similar finding was reported in other crop species such as tomato, pepper, rice, maize, cotton and soy bean etc. [45]. 5 g dosage of AM inoculum is sufficient for the maximum uptake other essential nutrients like Fe, Cu and Zn in cardamom seedlings. Given that Indian agricultural soils are deficient in Zn, mycorrhizal association can be an important tool to address this issue [46]. Several studies have demonstrated that mycorrhizal inoculation enhances Zn uptake in various crop species [47,48]. In contrast to our study, some researchers have found that mycorrhizal colonization can reduce manganese uptake in plants [49]. The increased uptake of these nutrients could be due to their absorption and mobilization by the fungi, which in turn enhances the uptake of these nutrients by plants. The influence of mycorrhizal fungi on nutrient uptake depends on complex interactions between the fungi, nutrients, and soil properties.

Soil enzyme activity can be used as an indicator of the functioning of soil ecosystems. These enzymes are involved in a range of soil processes, including nutrient cycling and organic matter decomposition [50]. According to previous reports, the high levels of enzymes activity observed in rhizosphere can be attributed to their secretion by either roots or fungi [51]. Soil phosphatases play a crucial role in soil P cycling and can be influenced by various factors, such as rhizosphere processes and crop plant growth stages [52]. Our study found that the AM treatments significantly affected with the high alkaline and acid phosphatase activity with a 10 g inoculum dose. This suggests that the increase in phosphatase activity in the rhizosphere soil could be attributed to a higher phosphatase release from mycorrhizal roots. Additionally, AM fungal inoculation may impact the soil microbial community, which plays a key role in determining the potential for enzyme synthesis [53]. Hence, to optimize plant nutrition, mycorrhizal inoculation must be carefully tailored to the individual crop and soil needs.

In addition to improving the soil physical, chemical and biological properties and also increasing the plant growth, the associated disease reduction mechanism imparted by AM fungi further adds feather to its caps. In the present study, it was noticed that inoculation of AM fungi to polybags reduced the disease incidence when compared to inoculated control and more specifically addition of 10 g to polybags significantly reduced the PDI. Similar results were reported by Wu et al. [54] upon inoculation of AM fungi to watermelon seedlings which showed reduced incidence of *R. solani* by alleviating the oxidative stress generated during host pathogen interaction. Evidences for reduction of soil borne pathogen *Pythium* spp. in presence of AM has been reported in several crops such as *Carica papaya* [55], *Cucumis sativus* [56], *Lycopersicon esculentum* [57] and in *Trifolium repens* [58]. Several evidences of reduction in *Fusarium* spp. infection upon inoculation of AM have been reported and it is mainly due to induction of systemic resistance in plants and thereby triggering the defense genes activation [[59], [60], [61]].

Our SEM results suggested that the mycorrhizal colonization is the indirect effects by spore count and mycorrhizal dependency via increasing number of fibrous roots. Spore count and mycorrhizal dependency strongly affected the AM colonization. Plant P uptake was directly influenced by alkaline phosphatase and mycorrhizal colonization. In *Camellia sinensis,* SEM analysis indicated that pathways of P metabolism, as well as the direct effect of AM inoculation, were of the most important causes in increasing root biomass [62]. Higher AM colonization enhances nutrient uptake by the plant, especially P [63].

## 5 Conclusion

Based on the findings from the two-year study evaluating different doses and application frequencies of arbuscular mycorrhizal (AM) fungal inoculum on cardamom seedlings, the 10 g dose was the most effective. It significantly increased shoot length, dry weight, and nutrient uptake, particularly phosphorus and calcium. This dose also reduced disease incidence and enhanced rhizosphere phosphatase activity. Additional sequential applications did not further reduce disease. The study suggests that using a 10 g dose of AM inoculum can enhance cardamom seedling growth, nutrient absorption, and disease resistance, providing a sustainable option for farmers. Future research should focus on assessing the effects of AM inoculation on cardamom yield and quality under field conditions, which could lead to more sustainable farming practices. Farmers can benefit from adopting AM inoculation to produce healthier seedlings, improve nutrient uptake, and decrease reliance on chemical fertilizers and pesticides, ultimately contributing to more resilient and productive agricultural systems.

## CRediT authorship contribution statement

**Sarathambal Chinnathambi:** Writing – review & editing, Writing – original draft, Resources, Methodology, Investigation, Data curation, Conceptualization. **Mohammed Faisal Peeran:** Resources, Methodology, Investigation. **Veeraraghavan Srinivasan:** Writing – review & editing, Resources, Data curation. **Sivasankaran Mukesh Sankar:** Writing – review & editing, Formal analysis, Data curation. **Priya George:** Writing – review & editing, Investigation, Formal analysis.

## Data availability statement

The authors confirm that the data supporting the findings of this study are available within the article.

## Funding

The work did not receive any funding.

## Declaration of competing interest

The authors declare that they have no known competing financial interests or personal relationships that could have appeared to influence the work reported in this paper.
