# Supplementary material for: Optimizing mycorrhizal fungi application for improved nutrient uptake, growth, and disease resistance in cardamom seedlings (Elettaria cardamomum (L.) Maton)
Source: Heliyon. 2024 Oct 10;10(20):e39227. doi: 10.1016/j.heliyon.2024.e39227 (PMC11620270; doi:10.1016/j.heliyon.2024.e39227)
Supplement: Multimedia component 1 [file mmc1.docx]

##################################################################################

##################### R Codes for Structural Equational Modelling (SEM) #################

##################################################################################

#Loading R packages

library(lavaan)

library(semPlot)

#Read the raw input data

Card<-read.csv("Card.csv", header=T)

# Compactly display the structure of data

str(Card)

#change the structure of the data

Card$AMF=as.numeric(Card$AMF)

Card$Seq=as.numeric(Card$Seq)

Card$Rep=as.factor(Card$Rep)

# Compactly display the structure of data

str(Card)

####################

#SEM Model fitting

sitemod <- 'P ~ DW + Colonization.

DW ~ FR + PH

PH ~ Mg

AMF ~ AlP

Colonization. ~ M_density + Spore_Count

AlP ~ Mg

FR ~ Colonization.+ AMF

Colonization.~ AMF

PH ~ FR

P ~ AlP

FR ~ Ca + Zn

####################

#Perform SEM analysis

sitemod.fit <- sem(sitemod,data=Card,missing="ML",fixed.x=FALSE,meanstructure = TRUE)

#Getting SEM analysis Summary

summary(sitemod.fit,standardized=TRUE,rsquare=T)

#Analyse the fit criteria of the model

fitMeasures(sitemod.fit,c("gfi","srmr"))

#Drawing Path diagram of the model

semPaths(sitemod.fit,

title = FALSE,

curvePivot = TRUE,

what = "std",

rotation = 2,

layout = "tree2",

optimizeLatRes = TRUE,

intercepts = FALSE,

edge.label.cex = 0.65,

exoVar=FALSE,

sizeMan=5,

sizeLat=3,

nCharNodes=10,

residuals=FALSE,

fixedStyle=1,

freeStyle=1,

curvePivot = FALSE)

############################# END OF ANALYSIS ######################################
